# *In vivo* and *in vitro* immune responses against *Francisella tularensis* vaccines are comparable among Fischer 344 rat substrains

**DOI:** 10.3389/fmicb.2023.1224480

**Published:** 2023-07-13

**Authors:** Roberto De Pascalis, Varunika Bhargava, Scott Espich, Terry H. Wu, H. Carl Gelhaus, Karen L. Elkins

**Affiliations:** ^1^Laboratory of Mucosal Pathogens and Cellular Immunology, Division of Bacterial, Parasitic and Allergenic Products, Center for Biologics Evaluation and Research, U.S. Food and Drug Administration, Silver Spring, MD, United States; ^2^Center for Infectious Disease and Immunity and Department of Internal Medicine, University of New Mexico, Albuquerque, NM, United States; ^3^MRIGlobal, Kansas City, MO, United States

**Keywords:** *Francisella tularensis*, tularemia, Fischer 344 rats, intracellular bacteria, vaccines

## Abstract

Identifying suitable animal models and standardizing preclinical methods are important for the generation, characterization, and development of new vaccines, including those against *Francisella tularensis*. Non-human primates represent an important animal model to evaluate tularemia vaccine efficacy, and the use of correlates of vaccine-induced protection may facilitate bridging immune responses from non-human primates to people. However, among small animals, Fischer 344 rats represent a valuable resource for initial studies to evaluate immune responses, to identify correlates of protection, and to screen novel vaccines. In this study, we performed a comparative analysis of three Fischer rat substrains to determine potential differences in immune responses, to evaluate methods used to quantify potential correlates of protection, and to evaluate protection after vaccination. To this end, we took advantage of data previously generated using one of the rat substrains by evaluating two live vaccines, LVS and *F. tularensis* SchuS4-Δ*clpB* (Δ*clpB*). We compared immune responses after primary vaccination, adaptive immune responses upon re-stimulation of leukocytes *in vitro*, and sensitivity to aerosol challenge. Despite some detectable differences, the results highlight the similarity of immune responses to tularemia vaccines and challenge outcomes between the three substrains, indicating that all offer acceptable and comparable approaches as animal models to study *Francisella* infection and immunity.

## Introduction

*In vitro* and *in vivo* preclinical studies are fundamental for advancing vaccine development. However, diseases such as tularemia, caused by *Francisella tularensis* (*Ft*), have a low incidence in nature (Staples et al., 2006). Correspondingly, clinical trials to establish vaccine efficacy are probably not feasible (Williams et al., 2019). The FDA “Animal Rule” provides a regulatory pathway to product approval by which efficacy may be established in animals and bridged to people (Snoy, 2010; Beasley et al., 2016; Allio, 2018). For products such as tularemia vaccines, the need for well-characterized animal models has been documented thoroughly. Moreover, animal studies provide an approach to evaluate immune responses against vaccines (Elkins et al., 2003, 2007; Conlan et al., 2011). Mice, particularly BALB/c and C57BL/6 strains, have been the animal models most utilized for evaluating tularemia vaccine efficacy (Nicol et al., 2021). However, BALB/c and C57BL/6 have Th2-and Th1-skewed immune responses, respectively, which may not be representative of human immune responses against *Ft* (Twine et al., 2012). In addition, protection in mice against aerosol Type A *Ft* is achieved only against relatively low challenge doses (Chen et al., 2003). This limits a full evaluation of vaccine efficacy, particularly when vaccines may have minimal differences (De Pascalis et al., 2022). To overcome these issues, we and others explored additional animal models that can mitigate these challenges.

*Ft*, a gram-negative bacterium, is classified as a Tier 1 bioterrorism agent due to its low infection dose, ability to be aerosolized, and severity of the disease it causes. Historically, the only vaccine under clinical investigation in the U.S. was live vaccine strain (LVS), which was established in the 1960s from the *Ft* subsp. *holarctica*, Type B (Eigelsbach and Downs, 1961; Saslaw et al., 1961a,b; Tigertt, 1962). LVS was investigated for its protection in laboratory workers primarily at the United States Army Research Institute of Infectious Disease (USAMRIID) (Burke, 1977). In people, LVS vaccination appears to offer partial protection against subsequent exposure to virulent Type A *Ft* (Saslaw et al., 1961a,b; Burke, 1977; Williams et al., 2019). For *Ft* vaccine studies, rabbits, which are a natural host of *Francisella*, appear to provide a better animal model compared to rodents to evaluate antibody immune responses (Shoudy et al., 2021); however, reagents for other immunological studies using rabbits are limited. Efforts in the last few years have characterized Fischer 344 rats as a model for the evaluation of medical countermeasures. The Fischer 344 rat was first described in 1981 as a tularemia model (Jemski, 1981). Neither LVS nor *Francisella novicida* are virulent in Fischer rats, similar to humans and in contrast to the high sensitivity of mice to LVS and *F. novicida*. In addition, rats can be challenged with the virulent Type A *Ft* SchuS4 (Ray et al., 2010) at higher doses than mice, providing a larger dynamic range to study vaccine efficacy. Moreover, rats vaccinated subcutaneously (s.c.) with LVS and SchuS4-Δ*clpB*-derived vaccines survive large intratracheal (i.t.) or aerosol challenge doses with highly virulent Type A *Ft* SchuS4 (De Pascalis et al., 2022). Finally, rats have the practical advantage of larger size compared to mice, allowing larger sample volumes to be collected for immunological assays; this facilitates the analysis of individual animals, in many cases without sacrificing the animal, and reduces overall the number of animals used for each study.

Because intracellular pathogens such as *Francisella* induce protective immune responses primarily via T cell-mediated immunity (Tärnvik et al., 1985; Bosio and Elkins, 2001; Elkins et al., 2011), we extended studies that were initiated in mice (De Pascalis et al., 2012, 2014, 2015, 2016), to Fischer 344 rats to identify correlates of vaccine-induced protection (De Pascalis et al., 2018). Initially, we used LVS-derived vaccines to identify correlates of protection, and later we used LVS as comparator to evaluate vaccines derived from Type A *Francisella* (De Pascalis et al., 2022). This approach took advantage of an *in vitro* co-culture method where *Francisella*-infected macrophages are used to re-stimulate leukocytes derived from differentially vaccinated rodents (Collazo et al., 2009; Elkins et al., 2011; De Pascalis et al., 2016, 2018, 2022). The *in vitro* functions measured with this assay allowed an initial screening to predict the efficacy of vaccine candidates, which could be selected for more extended studies in non-human primates. Using this approach, our previous studies suggested that at least two vaccines, SchuS4-Δ*clpB* (Δ*clpB*) and SchuS4-Δ*ClpB-*Δ*capB* may be vaccine candidates that are at least as good as, if not better than, LVS (De Pascalis et al., 2022).

During these studies, we identified at least three different rat substrains from vendors for laboratory studies, all named Fischer 344. By taking advantage of data previously generated using LVS and Δ*clpB* vaccines in one rat substrain (NHsd), in this study, we compared immune responses after primary vaccination with LVS and Δ*clpB*, adaptive immune responses upon re-stimulation of leukocytes *in vitro*, and the sensitivity to aerosol challenge of non-vaccinated and vaccinated animals from three Fischer 344 rat substrains. The goal of this comparative study was, therefore, to evaluate whether differences between these substrains impact tularemia vaccine evaluations.

## Materials and methods

### Experimental animals

Five-to-seven-week-old specific pathogen free male and female inbred Fischer rat, substrains F344/NHsd, F344/DuCrl, and F344/IcoCrl were purchased from Envigo (Indianapolis, IN), Charles River Laboratories (Wilmington, MA) and Charles River Laboratories (Italy and Germany), respectively. Rats were age and sex matched within each experiment. For studies performed at CBER/FDA, animals were housed 2 per cage in sterile cages with HEPA filtered circulating air. Rats were fed *ad libitum* with irradiated food and autoclaved chlorine dioxide-treated water. The room was maintained at negative pressure with temperature and humidity control as well as a 12 h light on/off cycle. For studies performed at UNMHSC, female rats were housed 5 per cages in individually ventilated cages (Techniplast, Italy) in an ABLS-2 laboratory before challenge and in a CDC-certified Select Agent ABSL-3 laboratory after challenge. The animal housing rooms were maintained under temperature control (73.0 ± 0.3°F in ABSL-2 and 70.5 ± 0.6°F in ABSL-3) with a 12 h light on/off cycle. The animals were fed *ad libitum* with Teklad irradiated rodent chow #2920x (Envigo) and given chlorine dioxide-treated filtered water. For studies performed at MRI Global, animals were group-housed 2 per cage in polycarbonate Techniplast caging, fed *ad libitum* (Harlan Teklad) and provided fresh tap water (supplied by Kansas City Municipality). The feed and water were free of substances reasonably expected to interfere with the objectives of the study. All rats were housed in environmentally controlled rooms, at a temperature of 68.0°F to 79.0°F and a relative humidity of 50% ± 20% with a 12 h light/dark cycle per day.

### Ethics statement

All experiments were performed under protocols approved by the Animal Care and Use committee of CBER (#2015-21), UNMHSC (#19-200,938-HSC) and MRI Global (AUS 16-33). These protocols meet the standards for humane animal care and use set by the Guide for the Care and Use of Laboratory Animals and U.S. Public Health Service policy. After any procedure, the health of the animals was monitored, and humane endpoints were observed. When naïve or immunized animals suffered an impairment of their health status and, or the indicated time points, the animals were euthanized with carbon dioxide inhalation in a euthanasia chamber where carbon dioxide was introduced at the rate of at least 30% of the chamber volume per minute or with sodium pentobarbital (Fatal-Plus; Vortech Pharmaceuticals, Dearborn, MI). As expected, the health of *Ft* challenged animals deteriorated within few days after challenge. The animals were euthanized when they were not able to move, even in response to physical stimulus, and therefore were unable to reach water and food, and the time of sacrifice recorded as the time of death.

### Bacteria and growth conditions

*Ft* LVS (American Type Culture Collection 29,684) and *Ft* Δ*clpB* [obtained from Conlan et al. (2010)] were grown to mid-log phase in modified Mueller-Hinton (MH) broth (Difco Laboratories, Detroit, MI), harvested, and frozen in aliquots in broth alone at−80°C (Baker et al., 1985; Fortier et al., 1991). *Ft* strain SchuS4 (BEI Resources, Manassas, VA, NR-10492) was sub-cultured in modified cysteine partial hydrolysate (MCPH) broth to produce a sub-master stock and a working stock, which were frozen in aliquots with 20% glycerol at −80°C. Of note, *Ft* SchuS4 NR-10492 has been demonstrated to be identical and with similar level of virulence compared to *Ft* SchuS4 NR-28534 (Lovchik et al., 2021), which was used previously to evaluate vaccine efficacy in NHsd rats (De Pascalis et al., 2022). Bacteria were periodically thawed for quality control by quantification of viability on MH agar plates or chocolate agar plates (Hardy Diagnostics, Santa Maria, CA); in addition, quantification was also evaluated at the time of vaccination or challenge procedures. Although different media were used at each site, within each laboratory’s work with the three rat strains, bacteria were cultured using the same media.

### Bacterial immunizations and challenge

Rats were anaesthetized with isoflurane and immunized by s.c. administration with 5 × 10^6^ to 1 × 10^7^ colony forming units (CFU) for each vaccine, diluted in 0.1 mL phosphate-buffered saline (PBS) (BioWhittaker/Lonza, Walkersville, MD). Control groups received PBS. Actual doses of administrated vaccines were determined by retrospective plate count. To evaluate sensitivity against primary aerosol challenge with *Ft* SchuS4 (performed at MRIGlobal), rats were weighed and randomized in 4 groups of 6 animals within each substrain, including both male and female, to receive different doses of *Ft* SchuS4. *Ft* strain SchuS4 was subcultured in cation-adjusted Mueller Hinton broth with 0.1% glucose and 2% IsoVitalex (MHB+). Cultures were diluted in MHB+ and aerosols were generated from the diluted cultures. Rats were placed in restraint tubes, and the tubes were loaded onto a CH Technologies aerosol chamber, where rats were exposed to aerosol for 10 min. The entire lung of each animal was then homogenized, and samples were serially diluted and plated on modified Thayer Martin (MTM) agar plates to determine the deposited dose. To evaluate vaccine efficacy (performed at UNMHS), the study animals were divided into multiple runs and exposed to aerosolized *Ft* SchuS4. SchuS4 was cultured for 18 h in Chamberlains Chemically Defined Medium (Teknova; Hollister, CA) and then diluted in brain heart infusion broth (Teknova) to the concentrations required to achieve the desired target presented dose and lung deposition based on historical correlations. Aerosols were generated using a Collison 3-jet nebulizer (BGI, Inc., Waltham, MA) and presented to rats in a nose-only exposure chamber (In-Tox Products, Inc., Moriarty, NM). For each run, an impinger sample was plated on chocolate agar (Hardy Diagnostics; Santa Maria, CA) to determine the aerosol concentration. The presented dose for each exposure run was calculated using the measured aerosol concentration, 20 mL impinger volume, 5 L/min flow rate through the impinger, 15 min exposure time, and the respiratory minute volume estimated using the Guyton formula and body weight. One rat was included in each exposure run and euthanized after all exposure runs for the day were completed; the lungs from this animal were homogenized and plated onto Thayer Martin Modified agar with vancomycin, colistin, nystatin, and trimethoprim (Hardy Diagnostics) to enumerate viable bacteria deposited in the lungs. The inclusion of antibiotics limits growth of contaminating lung bacteria and facilitates detection of *Ft* SchuS4. The LD_50_ of *Ft* SchuS4 for female NHsd rats was estimated to be approximately 1 CFU/rat, based on lung depositions (Hutt et al., 2017). Rats were monitored for survival for at least 21 days.

### Body weight and clinical observations

Body weights were recorded before challenge and every 2 to 3 days for the duration of the survival studies of 28 days. For the lethality study, body weights were not recorded post-challenge. In addition, the challenged animals were observed daily and assigned a clinical score from 0 to 5, with 0 reflecting no outward signs of illness; (1) mild clinical signs of infection such as coat ruffling and porphyrin secretion; (2) decreased activity or labored breathing; (3) no spontaneous movement but responds to stimulus such as touch or cage movement; (4) unresponsive to tail tug and euthanized; and (5) found dead. Observations were not blinded.

### Serum collection and PBL preparation

Two weeks after immunization, 300 μL of blood was collected from the tail vein of vaccinated rats using 21G blood collection sets (Terumo Medical Products; Somerset, NJ) into microcentrifuge tubes and centrifuged at 8500 *g* for 5 min at room temperature. The sera were transferred into fresh microcentrifuge tubes and stored at −20°C until use. After the serum samples were thawed, they were stored at 4°C until all analyses were completed. Alternatively, for gene expression analyses after immunization, blood was collected in heparin and used to prepare PBLs, which were stored in RNA*later* (Ambion, Austin, TX) at −80°C for further analyses.

### Evaluation of serum antibodies

ELISA plates (Thermo Scientific) were coated with 100 μL of 2 mg/mL purified *Ft* LPS (kindly provided by Dr. Wayne Conlan) in PBS pH 7.2 for 2 h at 37°C and then overnight at 4°C. The plates were blocked with 200 mL 0.85% NaCl with 0.05% Tween-20 and 10% FBS (blocking solution) for 30 min at 37°C. Serum samples and reference standards (pooled immune sera collected from 5 of 6 surviving NHsd rats approximately 14 days after s.c. injection with 10^3^ CFU *Ft* SchuS4 were serially diluted in blocking solution, and then 100 μL was added to each well for 1 h at room temperature). Bound anti-LPS IgG antibodies were detected using 100 μL of goat anti-rat IgG antibodies conjugated with horseradish peroxidase (Southern Biotech) in blocking solution for 1 h at room temperature. The plates were washed with 0.85% NaCl with 0.05% Tween-20 between each step. The plates were then developed using a solution of 3,3′,5,5′-tetramethylbenzidine (TMB; EMD Millipore) and the reaction was stopped with 0.5 N H_2_SO_4_. The optical density at 450 nm was read using microplate reader (BioTek) and Gen5 Data Analysis Software (BioTek). A standard curve was constructed using 4PL curve fit and was used to determine the titer of anti-LPS IgG antibodies in the serum samples as arbitrary units compared to reference sera.

### Co-culture of bone marrow derived macrophages with leukocytes

Five to-six weeks after immunization, 1 × 10^6^/wells of ACK-treated bone marrow derived cells from naïve rats were plated in 24 well plates and cultured in DMEM supplemented with 10% heat-inactivated FCS (HyClone, Logan, UT), 10 ng/mL rat CSF, 0.2 mM L-glutamine, 10 mM HEPES buffer, 1 mM sodium pyruvate, 1 mM sodium bicarbonate and 0.1 mM non-essential amino acids. During the first 24 h, cells were also treated with 50 μg/mL gentamicin. After 7 days of culture with media changes, confluent macrophages were infected for 2 h with LVS at a multiplicity of infection (MOI) of 1:50, then washed, treated with 50 μg/mL gentamicin, and washed (Rice et al., 2020). Splenocytes and PBLs prepared from naïve and vaccinated animals were treated with ACK lysing buffer (BioWhittaker/Lonza), washed with PBS 2% FCS, assessed for viability by exclusion of trypan blue, and co-cultured with LVS-infected macrophages at 5 × 10^6^ cells/well. To obtain PBLs for the co-culture studies, blood was pooled from 3–4 animals for each vaccine group. After 2 days of culture, non-adherent cells were recovered, pelleted, and stored in RNA*later* at −80°C. Supernatants were also collected and stored at −80°C, and adherent LVS-infected macrophages were lysed to determine LVS intramacrophage replication by plate count.

### Real time PCR

Approximately 1 × 10^6^–1 × 10^7^ of PBLs were prepared from blood, and PBLs and splenocytes were recovered from co-cultures, and stored in RNA*later* at −80°C. Cell preparations were used to purify total RNA using RNeasy mini kits (Qiagen, Valencia, CA), according to the manufacturer’s instructions. Up to one microgram of RNA was used to synthesize cDNA (High-Capacity RNA-to-cDNA) (Applied Biosystems, Carlsbad, CA), following the manufacturer’s instructions. Semi-quantitative real-time PCR amplification of a panel of 19–23 genes of immunologic interest plus Gusb, RPS29, and GAPDH as housekeeping genes, all from Applied Biosystems (Carlsbad, CA), was completed with a ViiA 7 Real Time PCR system (Applied Biosystems). The panel of genes included factors that were previously identified as correlates of protection against *Ft* (De Pascalis et al., 2022) or based on the outcomes obtained in previous studies (De Pascalis et al., 2016, 2018). Additional genes that typically showed minimal differential expression, such as TNF-α, IL-6, and Csf2, were also included in the panel. After normalization, delta *Ct* (Δ*Ct*) and the ratio between Δ*Ct* of vaccine samples and control samples (PBS treated) were calculated (ΔΔ*Ct*), which allows a semi-quantitative analysis (relative to PBS control) of gene regulation.

### Assessment of supernatants

IFN-γ and nitric oxide production were assessed in the supernatants recovered from the *in vitro* co-cultures. Estimation of nitric oxide (NO) was performed using the Griess reaction (Sigma-Aldrich, St Louis, MO) (Green et al., 1982) and serially diluted NaNO_2_ as reference. Quantification of IFN-γ was assessed using standard sandwich ELISAs and the recombinant standard protein (BD Pharmingen, San Diego, CA) as comparator, according to the manufacturer’s instructions.

### Statistical analyses

Microsoft Excel and GraphPad software were used to evaluate differences in bacterial growth, IFN-γ and NO production, relative gene expression, and antibody titers. Comparisons were evaluated by one-way ANOVA with Tukey’s multiple comparisons test. CFU data were log_10_ transformed and cytokine concentrations were measured using a log scale; thus, a normal distribution was assumed. Significant differences were evaluated using a two-tailed student’s *t*-test, with a *p*-value of <0.05 indicating significance. Probit analysis was used to evaluate the survival of naïve animals against aerosol challenge with *Ft* SchuS4. Comparisons of survival curves between different groups of rats were statistically evaluated by Kaplan–Meier and log-rank (Mantel–Cox) analyses (GraphPad Software, Version 9.1.1, San Diego, CA, United States).

## Results and discussion

### Serum antibody responses in Fischer 344 rats substrains vaccinated with LVS and Δ*clpB*

**Figure 1:**
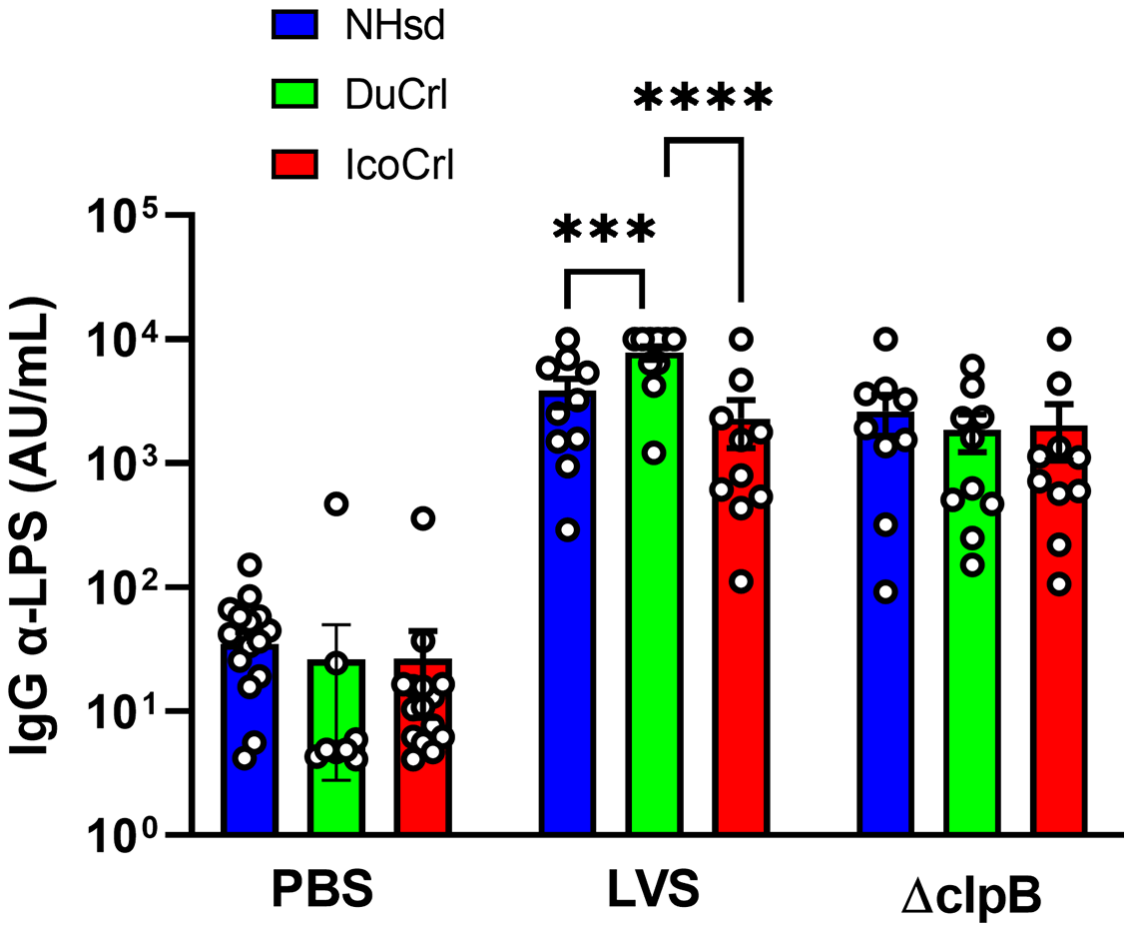
Serum anti-*Francisella* antibody titers from vaccinated rats show minimal differences between rat substrains. Fischer rats were vaccinated as indicated. Two weeks after vaccination, serum samples were collected and analyzed for anti-LPS IgG. Shown are the results from two independent studies. Values indicate the mean titers obtained from serum from ten rats per group. Error bars indicate the standard error of the mean (s.e.m.). Brackets indicate statistical significance (^***^ = *p* < 0.001, ^****^ = *p* < 0.0001).

To evaluate humoral immune responses afforded by Δ*clpB* and LVS after primary vaccination, F344 rats substrains were immunized with these live attenuated vaccines. Two weeks after vaccination, we obtained sera from ten individual animals per vaccine substrain group, and we then determined relative quantities of IgG anti-LPS serum antibodies. Serum antibodies were readily detectable in all vaccinated rats (Figure 1). We found substantial animal-to-animal variability. Among the LVS-vaccinated animals, the DuCrl substrain produced slightly and significantly higher amounts of anti-LPS IgG compared to either the NHsd or IcoCrl substrains. The biological significance of this subtle difference is not clear, particularly since no obvious differences in mean levels were apparent between rat substrains vaccinated with Δ*clpB*.

### Relative gene expression in blood after vaccination

**Figure 2:**
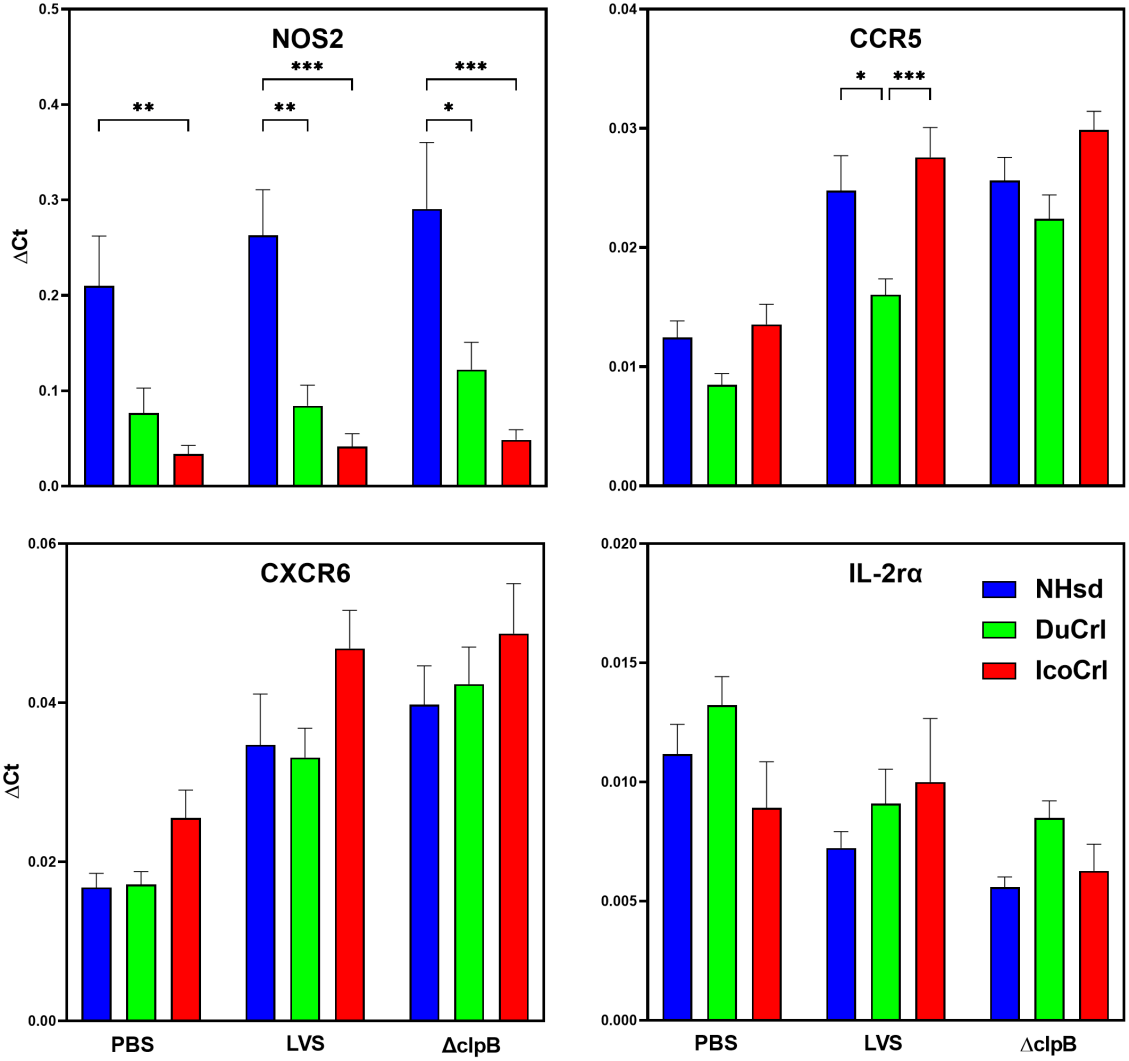
PBLs from vaccinated rats show similar patterns of relative gene expression between rat substrains. Fischer rats were vaccinated as indicated. Blood was collected 2 weeks after vaccination. PBLs were prepared and analyzed for relative gene expression. Semi-quantitative analyses of gene expression were performed using independent primers and probes as described in Materials and Methods. Shown are the results from two independent studies with a total of 10 animals per group. Values indicate mean of the Δ*Ct* and standard error of the mean (s.e.m.). Brackets indicate significant differences (^*^ = *p* < 0.05; ^**^ = *p* < 0.01; ^***^ = *p* < 0.001). Shown are data from selected genes that exhibited different expression patterns between rat substrains.

To further evaluate potential differences among rat substrains after primary vaccination, the relative expression of a panel of immune-related genes was evaluated in PBLs 2 weeks after immunization. This panel was selected based on outcomes in previous studies of similar design. We also included genes potentially involved in T cell immune responses that previously exhibited minimal differences between vaccine groups. The normalized values (Δ*Ct*) of gene expression indicated relatively minor differences among substrains (Figure 2; Supplementary Figure S1). Some genes, such as Nos2, appeared to be upregulated in a pattern NHsd > DuCrl > IcoCrl. The expression of other genes, such as CCR5, was lower overall in PBLs from vaccinated DuCrl rats compared to those from the NHsd and IcoCrl rats, while others such as CXCR6 were higher in the IcoCrl substrain. These patterns were observed also with the PBS-injected animals; in few cases, notably Nos2, the differences were significant. The latter results suggested some inherent baseline differences between rat substrains. Further, at this time point the expression of about half of tested genes was higher in the LVS and Δ*clpB* groups across all the rat substrains, or lower, in the case of IL-2RA. To evaluate whether the differences observed between rat substrains affected relative gene expression after LVS or Δ*clpB* vaccination, the ΔΔ*Ct*, indicating the fold changes differences in comparison to the PBS group, was calculated (Table 1). The pattern of up-, down-or no-differential regulation mostly overlapped and were similar across rat substrains, and few differences were noticeable. Consistent with previous results, a number of genes were upregulated in cells from vaccinated rats compared to PBS-treated rats and trended toward more upregulation in PBLs from Δ*clpB*-vaccinated rats compared to those from LVS-vaccinated animals across all three rat substrains (Supplementary Table S1). In a few cases the differences in the degree of upregulation, such as for CCL5, TBET, IL12rβ2, and CXCL9, were significant (*p* < 0.05). Taken together, these results suggest that, although different animal backgrounds may have subtle effects on the amounts of gene expression, those differences do not interfere with detecting differential gene expression that reflects immune responses against vaccines.

**Table 1:** Relative gene expression of immune-related factors in PBLs 2 weeks after vaccination.

	LVS			Δ*clpB*		
	NHsd	DuCrl	IcoCrl	NHsd	DuCrl	IcoCrl
IFN-γ	3.0^^^ ± 0.6	2.4 ± 0.5	1.7^^^ ± 0.4	3.5 ± 0.9	2.8 ± 0.6	2.1 ± 0.3
NOS2	1.3 ± 0.2	1.1 ± 0.3	1.2 ± 0.4	1.4 ± 0.3	1.6 ± 0.4	1.4 ± 0.3
IL-21	1.2 ± 0.1	1.2 ± 0.1	1.6 ± 0.4	1.0^*,^^ ± 0.08	1.3^*^ ± 0.2	1.3^^^ ± 0.2
IL-18bp	1.1 ± 0.2	1.1 ± 0.07	1.3 ± 0.3	1.1^*,^^ ± 0.2	1.9^*^ ± 0.1	1.8^^^ ± 0.2
FASLG	1.6 ± 0.3	1.3 ± 0.2	2.2 ± 0.5	1.9 ± 0.4	1.5^#^ ± 0.1	2.4^#^ ± 0.3
SOCS1	1.4^*^ ± 0.2	0.8^*^ ± 0.2	1.2 ± 0.2	0.9 ± 0.2	0.9 ± 0.2	0.9 ± 0.2
GZMB	1.5 ± 0.3	1.6 ± 0.3	1.9 ± 0.5	2.0^*^ ± 0.3	1.3^*^ ± 0.1	1.6 ± 0.3
HMOX1	0.8 ± 0.1	1.0 ± 0.1	0.9 ± 0.2	1.1 ± 0.2	1.3 ± 0.1	1.1 ± 0.1
CCR3	0.7^^^ ± 0.1	1.1 ±0.2	1.2^^^ ±0.3	0.6^*^ ± 0.06	1.0^*^ ± 0.07	0.9 ± 0.2
CCR2	1.0 ± 0.2	0.8 ± 0.2	0.7 ± 0.1	1.2 ± 0.2	1.4 ± 0.1	1.0 ± 0.1
CCR5	2.0 ± 0.2	1.9 ± 0.2	2.0 ± 0.2	2.1^*^ ± 0.2	2.6^*^ ± 0.2	2.2 ± 0.1
CCL5	3.2^*^ ± 0.4	4.3^*^ ± 0.3	3.5 ± 0.4	4.8 ± 0.6	4.9 ± 0.6	5.4 ± 0.4
IL-12rβ2	2.0^*^ ± 0.3	2.7^*,#^ ± 0.1	2.2^#^ ± 0.2	3.0 ± 0.4	3.5 ± 0.4	3.6 ± 0.4
TBET	2.5^*^ ± 0.3	3.7^*^ ± 0.3	3.2 ± 0.3	3.5 ± 0.4	4.2 ± 0.6	4.4 ± 0.5
CXCR6	2.1 ± 0.4	1.9 ± 0.2	1.8 ± 0.2	2.4 ± 0.3	2.5 ± 0.3	1.9 ± 0.2
CXCl11	2.2^*^ ± 0.4	1.3^*,#^ ± 0.3	2.7^#^ ± 0.7	2.9^^^ ± 0.3	2.7^#^ ± 0.4	5.0^^,#^ ± 1.1
CXCl9	2.2 ± 0.5	2.1 ± 0.5	4.2 ± 1.2	5.4^^^ ± 0.4	4.9^#^ ± 0.5	13.2^^,#^ ± 3.4
LTA	1.5^*^ ± 0.2	0.9^*^ ± 0.1	1.5 ± 0.3	1.5 ± 0.2	1.3^#^ ± 0.2	1.9^#^ ± 0.3
IL-2RA	0.6 ± 0.1	0.7 ± 0.1	1.1 ± 0.3	0.5 ± 0.04	0.6 ± 0.1	0.7 ± 0.1

Gene expression analyses were performed using PBLs as shown in Figure 2. Fold changes were calculated in comparison to mean of naïve cells from the matching rat substrain. Shown are the means of fold change ± standard error of the mean (s.e.m.) calculated from 7–10 animal per group. Whitin each vaccine group, ^*^indicates significant differences between NHsd and DuCrl, ^^^indicates significant differences between NHsd and IcoCrl, whereas ^#^indicates significant difference between DuCrl and IcoCrl substrains (*p* < 0.05).

### *In vitro* re-stimulation of leukocytes from vaccinated rats

**Figure 3:**
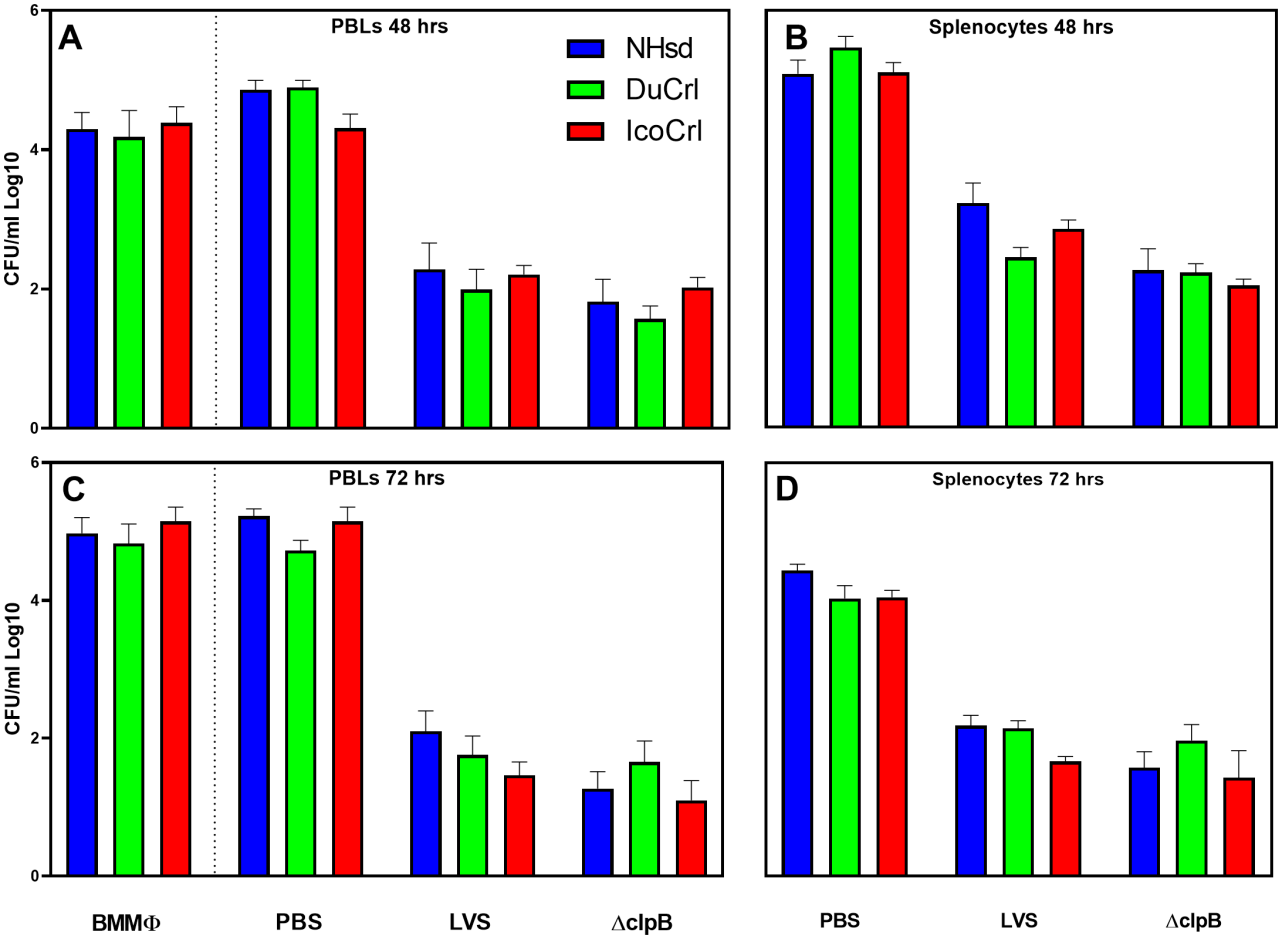
Rat leukocytes control intramacrophage bacterial replication *in vitro* in similar patterns between rat substrains. Fischer rats were vaccinated as indicated. Bone marrow-derived macrophages (BMMΦ) from three rat substrains were infected with LVS and co-cultured with the homologous PBL **(A,C)** or splenocytes **(B,D)** for 48 h **(A,B)** or 72 h **(C,D)**. BMMΦ were then lysed and colony forming units (CFU) determined. Values shown are the average from 2–3 independent experiments of similar design. Error bars indicate standard error of the mean (s.e.m.). *p*-values were calculated among rat substrains, within each vaccine group. None of the differences between rat substrains within vaccine group with different sources of cells were significant.

We next evaluated adaptive immune responses by measuring *in vitro* functions. We evaluated the functions of leucocytes *in vitro* by using a co-culture assay. This approach, which restimulates T cells, was developed in mice (Collazo et al., 2009; Elkins et al., 2011), adapted and modified in rats (De Pascalis et al., 2018; Rice et al., 2020), and successfully used to screen new vaccines (De Pascalis et al., 2022) against *Ft*. Here, leukocytes derived from PBS-treated or vaccinated animals were re-stimulated by co-culture with LVS-infected macrophages. We first evaluated the ability of leukocytes to control intramacrophage bacterial replication (Figure 3). Cells derived from all types of LVS-and Δ*clpB*-vaccinated rats controlled bacterial replication, while leukocytes from the PBS-treated group did not. We did not observe any significant differences in control of replication by PBLs or splenocytes between the rat substrains within each vaccine group. We then measured levels of mediators in supernatants recovered from co-cultures. The amounts of NO and IFN-γ production were inversely related to numbers of CFU recovered from infected macrophages (Supplementary Figure S2) after 48 h of co-culture (Panels A,C,E,G) and after 72 h (Panels B,D,F,H); no IFN-γ was detected in the supernatants of co-cultures containing naïve PBLs or splenocytes. Although we found variability across experiments, co-cultures containing leukocytes from all LVS-or Δ*clpB*-vaccinated rats produced large amounts of NO and IFN-γ. A trend toward slightly lower NO production in samples from vaccinated NHsd rats was observed, which was significantly different compared to that of IcoCrl in PBLs at 72 h. However, most of the differences were not significant, and we did not identify any obvious patterns across the substrains within each vaccine group at either 48-or 72 h of co-culture.

To further evaluate *in vitro* functions, the relative expression of 23 genes of immunologic interest was evaluated in PBLs and splenocytes recovered after 2 days of co-cultures (Table 2). This panel of genes included working correlates of protection previously developed using NHsd rats (De Pascalis et al., 2018). In addition, similar to the studies described above using PBLs obtained after vaccination without re-stimulation, we included genes not usually differentially expressed in recovered leukocytes from LVS-or Δ*clpB*-vaccinated NHsd rats. We compared the relative expression of these genes in cells from NHsd rats to those derived from the DuCrl and IcoCrl substrains, expressed as fold change compared to control cells. With a few exceptions, the majority of gene expression exhibited similar patterns in all rat substrains in both PBLs and splenocytes from LVS-or Δ*clpB*-vaccinated animals. However, in a few cases the degree of upregulation, either in PBLs or splenocytes, was different and significant between rat substrains. These included relative expression of IFN-γ, granzyme B, and IL-21 (Table 2). Notably, as previously demonstrated (De Pascalis et al., 2022), many genes were upregulated in leukocytes derived from Δ*clpB*-vaccinated rats, in amounts that were comparable to those in cells from LVS-vaccinated rats (Supplementary Table S2); in few cases the differences were significant (*p* < 0.05). Also of note, previously we used C57BL/6 mice and NHsd rat to identify genes whose expression correlated with *in vivo* protection of LVS-derived vaccines against *Ft* (De Pascalis et al., 2012, 2016, 2018). Here, we found a good correlation between the reference data from NHsd substrain and data shown here using DuCrl and IcoCrl substrains. Collectively, therefore, the results indicated that most genes were upregulated in patterns that were not influenced by rat substrain.

**Table 2:** Relative gene expressionof immune-related factors in rat PBLs and splenocytes recovered from co-cultures.

	LVS			Δ*clpB*		
	NHsd	DuCrl	IcoCrl	NHsd	DuCrl	IcoCrl
PBL						
CCL5	3.1 ± 0.5	3.4 ± 1.2	2.6 ± 0.6	5.5 ± 0.2	7.6 ± 0.9	6.3 ± 0.6
CCR3	1.05 ± 0.02	1.9 ± 1.1	0.9 ± 0.1	1.8 ± 0.2	4.4 ± 3.3	1.9 ± 0.9
CCR5	0.9 ± 0.1	0.8 ± 0.3	0.6 ± 0.1	1.0 ± 0.2	0.9 ± 0.03	0.7 ± 0.3
CXCL9	14.1 ± 11.2	10.1 ± 8.7	10.9 ± 6.0	5.1 ± 1.5	16.7 ± 15.7	9.3 ± 0.3
CXCR6	2.2 ± 0.4	2.8 ± 0.8	2.3 ± 0.4	3.6 ± 0.9	4.4 ± 1.0	6.9 ± 1.1
FASLG	1.8^*^ ± 0.03	2.3^*^ ± 0.1	1.3 ± 0.6	2.0 ± 0.02	2.9 ± 0.3	1.5 ± 0.6
GZMB	2.4 ± 0.6	1.7 ± 0.1	4.2 ± 1.1	2.3 ± 0.2	1.8 ± 0.4	3.0 ± 1.6
HMOX1	8.9 ± 6.1	5.3 ± 4.9	2.6 ± 0.3	2.7 ± 0.02	7.2 ± 6.7	1.9 ± 1.1
IFN-γ	4.9^*^ ± 0.8	6.0 ± 2.4	11.3^*^ ± 0.8	4.6 ± 1.5	7.9 ± 4.5	15.5 ± 9.3
IL-2RA	3.4 ± 0.03	4.2 ± 1.7	3.3 ± 1.8	2.2 ± 0.1	3.3 ± 1.4	2.5 ± 1.2
IL-12rβ2	3.2 ± 0.3	3.5 ± 1.3	3.5 ± 0.2	3.1 ± 0.2	4.0 ± 1.2	2.9 ± 0.7
IL-18bp	4.4 ± 1.5	3.4 ± 2.4	3.4 ± 2.2	2.7 ± 0.7	4.4 ± 3.3	3.6 ± 1.5
IL-21	30.1 ± 8.9	23.5 ± 2.9	53.1 ± 33.2	18.7^*^ ± 2.5	16.4 ± 5.7	31.8^*^ ± 1.6
LTA	10.0 ± 2.7	8.4 ± 2.8	10.7 ±2.8	6.6 ± 0.9	5.9 ± 1.5	10.1 ± 1.0
NOS2	10.4 ± 2.4	3.6 ± 1.9	16.9 ± 2.8	10.3 ± 6.9	6.1 ± 4.4	16.6 ± 12.7
SOCS1	1.9 ± 0.9	1.4 ± 0.5	3.5 ± 1.6	1.7 ± 0.4	1.2 ± 0.4	4.5 ± 2.6
TBET	1.9 ± 0.8	1.9 ± 0.7	2.4 ± 0.9	2.4 ± 0.4	1.5 ± 1.7	3.8 ± 2.2
IL-6	0.5 ± 0.2	0.5 ± 0.3	0.9 ± 0.5	0.4 ± 0.08	0.3 ± 0.2	1.0 ± 0.5
CSF2	1.6 ± 1.0	1.3 ± 0.9	2.8 ± 1.0	0.9 ± 0.3	0.9 ± 0.8	1.8 ± 0.7
CXCL11	0.5^*^ ± 0.01	0.2^*^ ± 0.03	1.4 ± 1.3	0.3 ± 0.1	0.2 ± 0.1	1.5 ± 1.4
CCR2	1.3^*^ ± 0.09	2.0^*^ ± 0.2	2.0 ± 0.4	2.2^*^ ± 0.3	2.8 ± 0.5	3.6^*^ ± 0.4
TNFα	1.6 ± 0.4	1.0 ± 0.2	2.3 ± 0.5	1.5 ± 0.3	0.8 ± 0.2	2.4 ± 0.6
PRF1	1.7 ± 0.4	1.6 ± 0.3	2.1 ± 0.02	2.1 ± 0.02	1.5 ± 0.5	2.3 ± 1.5
**Spleen**						
CCL5	1.3 ± 0.2	1.7 ± 0.01	1.3 ± 0.01	3.0 ± 0.2	4.3 ± 1.3	3.4 ± 0.3
CCR3	0.8 ± 0.1	0.9 ± 0.06	0.5 ± 0.05	1.4 ± 0.3	1.5 ± 0.3	1.6 ± 0.6
CCR5	1.6 ± 0.04	1.8 ± 0.4	1.6 ± 0.3	2.4 ± 0.1	2.4 ± 1.0	1.8 ± 0.5
CXCL9	4.0 ±0.9	5.6 ± 2.2	4.1 ± 1.8	6.6 ± 1.8	7.5 ± 4.5	4.2 ± 0.3
CXCR6	0.9 ± 0.2	1.1 ± 0.04	1.0 ± 0.03	1.9 ± 0.4	1.6 ± 0.4	1.3 ± 0.03
FASLG	1.7 ± 0.08	1.9 ± 0.1	2.1 ± 0.1	2.6 ± 0.3	2.8 ± 0.3	2.3 ± 0.05
GZMB	3.6^*^ ± 1.3	2.9^^^ ± 0.02	8.0^*,^^ ± 0.01	4.5^*^ ± 1.3	5.3^^^ ± 0.4	8.3^*,^^ ± 0.1
HMOX1	1.3 ± 0.4	2.4 ± 0.9	1.7 ± 0.7	2.0 ± 0.1	4.1 ± 1.9	2.3 ± 1.0
IFN-γ	13.1 ± 3.9	18.0 ± 6.5	21.6 ± 5.3	14.8 ± 3.1	19.6 ± 12.9	16.9 ± 8.4
IL-2RA	1.8 ± 0.08	3.0 ± 0.8	3.5 ± 0.6	2.0 ± 0.3	2.2 ± 0.9	2.0 ± 0.3
IL-12rβ2	2.5 ± 0.3	3.7 ± 0.6	5.0 ± 0.5	3.5 ± 0.3	3.9 ± 1.6	4.1 ± 0.8
IL-18bp	1.5 ± 0.07	1.6 ± 0.07	1.7 ± 0.06	2.2 ± 0.3	2.1 ± 0.8	1.8 ± 0.01
IL-21	12.8^*^ ± 2.4	30.7^*^ ± 3.4	42.7 ± 2.8	11.9 ± 1.2	19.8 ± 6.6	14.8 ± 2.3
LTA	6.5 ± 1.6	10.9 ± 3.7	13.9 ± 3.0	7.8 ± 2.4	8.9 ± 5.2	9.9 ± 2.1
NOS2	2.6 ± 0.5	3.6 ± 1.6	4.3 ± 1.3	4.2 ± 1.5	4.8 ± 2.6	4.2 ± 0.2
SOCS1	2.8 ± 0.4	3.8 ± 0.04	3.9 ± 0.03	3.6 ± 0.4	2.4 ± 0.6	2.9 ± 0.8
TBET	1.7^*^ ± 0.03	2.1^*^ ± 0.04	2.2 ± 0.03	2.2 ± 0.1	2.1 ± 0.5	2.1 ± 0.1
IL-6	0.7 ± 0.3	0.5 ± 0.09	0.5 ± 0.07	0.6 ± 0.2	0.4 ± 0.06	0.5 ± 0.2
**Spleen**						
CSF2	1.2 ± 0.4	1.2 ± 0.01	1.3 ± 0.01	0.8 ± 0.2	0.6 ± 0.07	0.6 ± 0.2
CXCL11	1.6 ± 0.5	1.9 ± 0.4	1.6 ± 0.3	3.5 ± 1.6	1.9 ± 0.6	1.6 ± 0.6
CCR2	1.7 ± 0.5	2.0 ± 0.5	1.8 ± 0.4	2.9 ± 0.8	2.9 ± 1.3	2.8 ± 0.2
TNFα	1.5 ± 0.4	1.5 ± 0.2	1.8 ± 0.2	1.8 ± 0.6	1.2 ± 0.3	1.6 ± 0.02
PRF1	1.4 ± 0.2	1.0 ± 0.04	2.1 ± 0.04	1.6 ± 0.2	1.5 ± 10.1	2.4 ± 0.4

Gene expression analyses using cells recovered from in vitro co-cultures after 48 h were performed as described in Material and Methods. Fold changes were calculated in comparison to naïve cells (semi-quantitative analysis) and means of fold change ± standard error of the mean (s.e.m.) were calculated from 2 or 3 independent experiments. Within each vaccine groups, ^*^indicate significant differences (*p* < 0.05) between NHsd and DuCrl or between NHsd and IcoCrl substrains, whereas ^^^indicate significant difference between DuCrl and IcoCrl.

### *Ft* SchuS4 aerosol challenge of naïve and vaccinated rats

To evaluate susceptibility and protection of rat substrains against *Ft* SchuS4 aerosol challenge, we performed two sets of experiments. In the first test, we challenged naïve male and female NHsd and DuCrl substrains with different doses of *Ft* SchuS4 via aerosol (Table 3). The average weight of the animals used in this study were 125 g and 121 g for the NHsd and DuCrl substrains, respectively. IcoCrl animals from Europe were not available at the time this study was performed. Animals were observed twice daily. Signs of infection included rough coat, hunched posture, squinty eyes, lethargy, and debris around the eyes (porphyrin staining). Breathing abnormalities and nasal discharge were occasionally observed. The onset of symptoms occurred sooner in the higher dose groups, but no symptoms were observed before day 4 post-infection. There was no apparent difference in symptoms or survival across the two substrains. Most deaths occurred between days 5 and 10, although sporadic deaths occurred until day 22. At the highest dose all rats succumbed, whereas at the lowest dose, either 50% or 66% of rats survived. A probit analysis was conducted on the survival data to establish the susceptibility of both substrains against *Ft* SchuS4 aerosol challenge (Supplementary Figure S3). The LD_50_ was estimated to be 5 and 9 CFU for the NHsd and DuCrl substrain, respectively; the differences between the NHsd and DuCrl strains were within the 95% confidence interval and not considered significant. Similarly, no differences were apparent between male and female rats. It should be noted that calculations of aerosol dosing are imprecise at values below 10 CFU per animal and comparison of lethality curves is appropriate. Therefore, we observed comparable outcomes in these two substrains after primary *Ft* SchuS4 challenge.

**Table 3:** Evaluation of survival in naïve Fischer 344 substrains against *Ft* SchuS4 aerosol challenge.

Sub-substrain	Number of rats	Presented dose (CFU)	Deposited dose (CFU)	Percent survival
NHsd	6	66	6.8	50
	6	371	42.3	33.3
	6	5,142	204.5	0
	6	78,741	6,995	0
DuCrl	6	66	6.8	66.7
	6	371	42.3	0
	6	5,142	204.5	16.6
	6	78,741	6,995	0

Rats were randomly divided in groups of six and infected with *Ft* SchuS4 via aerosol, as described in Materials and Methods. Presented doses represent the average of four challenge runs; deposited doses represent the average calculated from two rats from four challenge runs. Rats were monitored for 28 days.

We then evaluated the *in vivo* protection of vaccinated animals against aerosol challenge with *Ft* SchuS4. All substrains were vaccinated as described above. Six weeks after vaccination, rats were challenged with fully virulent *Ft* SchuS4 by aerosol using two different challenge doses. The averages of presented doses from seven runs were 27,400 ± 7,400 CFU/rat (low dose) and 68,500 ± 1,800 CFU/rat (high dose). In this second set of experiments, the average weight of the animals used in the two survival studies were 193 g, 166 g, and 174 g, for the NHsd, DuCrl and IcoCrl substrains, respectively. Challenged animals were monitored for 21 days for survival. During this period, the body weight of all animals declined within 3–8 days after challenge regardless of vaccine used. Thereafter, animals either succumbed, or they regained weight and survived (Supplementary Figure S4). Overall, weight loss appeared slightly more evident in rats vaccinated with LVS followed by low dose challenge compared to the rats vaccinated with Δ*clpB*. However, no obvious or significant differences were observed either across vaccines or between rat substrains.

In addition, during this timeframe, the animals were observed daily to evaluate clinical signs of infection, which were recorded and scored from 0 to 5 (Supplementary Figure S5). Within 3–6 days, the health of control rats treated with PBS deteriorated and thereafter the animals succumbed. Except for those rats that succumbed (score of 5), all other vaccinated rats scored 3 or less. We observed a similar profile among the animals vaccinated with LVS and challenged at low and high doses, as well as the rats vaccinated with Δ*clpB* and challenged with a high dose. However, rats vaccinated with Δ*clpB* and challenged with the low dose exhibited better clinical scores compared to all others. Thus, similar to the body weight evaluations, we did not observe any obvious differences in clinical scores between rat substrains. Because these clinical evaluations were unblinded, statistical analyses were not performed.

**Figure 4:**
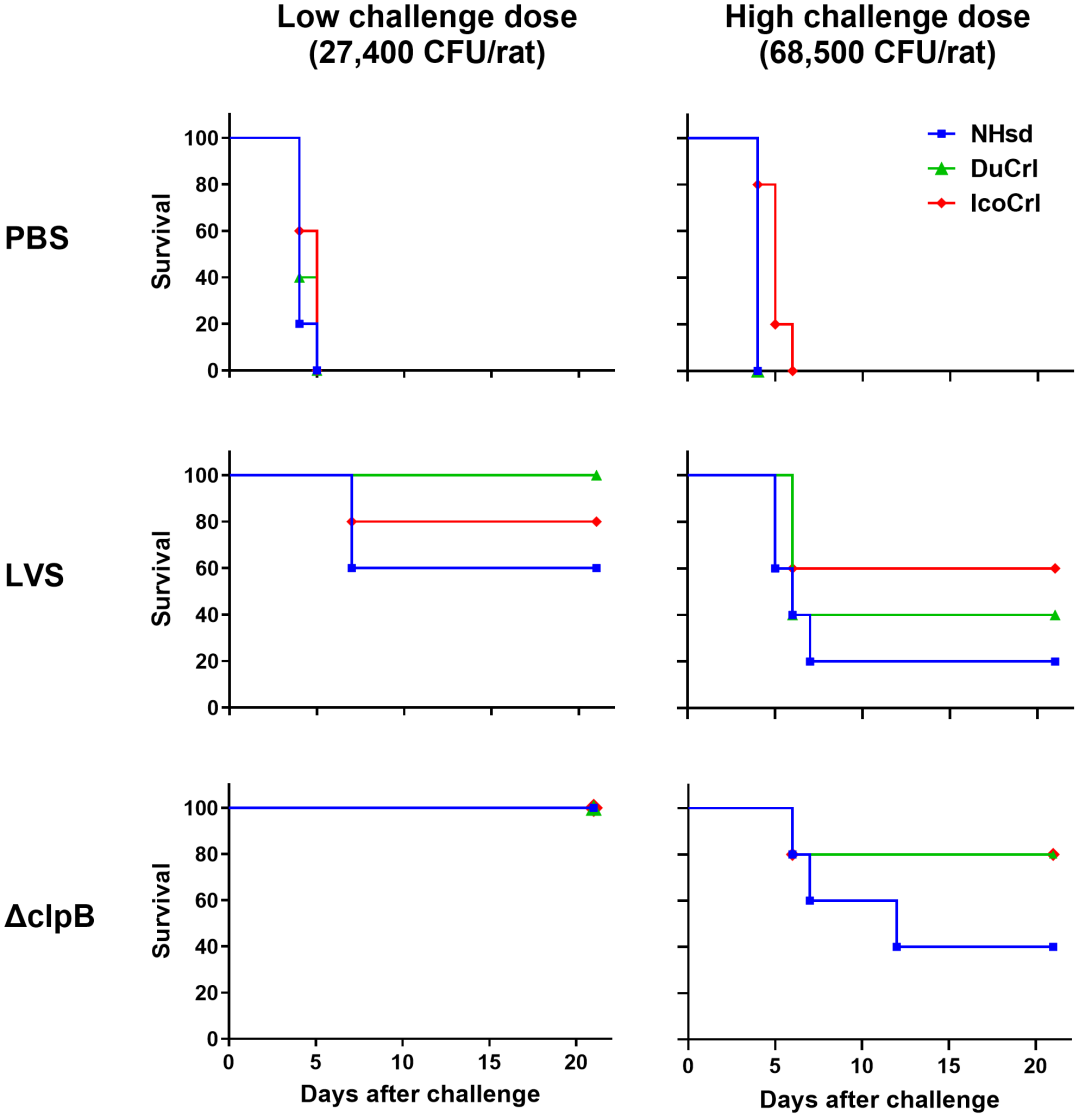
Survival outcomes of vaccinated rats after aerosol challenge with *Ft* demonstrated minimal differences between substrains. Fischer 344 rats were vaccinated as indicated. Six weeks after vaccination, rats were challenged by aerosol either with a high or low dose of *Ft* SchuS4. Survival was monitored for 21 days. The experiment included a total of 5 animals per vaccine-challenge dose group per substrain. For each challenge dose, bacterial lung deposition was monitored. The figure depicts time to death. Analyses of survival outcomes, calculated by Kaplan–Meier and log-rank (Mantel–Cox), indicated that differences between rat substrains were not significant. Whitin each challenge dose group, pairwise comparisons of LVS and Δ*clpB* by rat substrain showed no significant differences.

As noted, all rats treated with PBS died 3 to 6 days after challenge (Figure 4). As expected, more animals in all vaccinated groups survived when given a lower challenge dose compared to those challenged with a higher dose. Moreover, more animals vaccinated with Δ*clpB* survived compared to those vaccinated with LVS when subjected to either low or high challenge doses, and all rats vaccinated with Δ*clpB* and challenged with a low dose survived, independently of substrain. When we compared the survival outcomes between the rat substrains, the levels of protection for the DuCrl and IcoCrl substrains were similar to each other. NHsd rats vaccinated with either LVS or Δ*clpB* exhibited slightly less survival compared to the other two substrains. However, a pairwise comparison between the two vaccines, performed within each rat substrain, revealed that the differences were not significant. In addition, since the three rat substrains appeared to be very similar, we combined data from the three substrains for each vaccine and compared the results using a log-rank test. At the low challenge dose (*p* = 0.0726) and at the high challenge dose (*p* = 0.098), the differences were not significant. Taken together, as expected based on previous studies (De Pascalis et al., 2022), we observed subtle differences between vaccines: especially when using the low challenge dose, rats vaccinated with LVS and challenged appeared to be sicker overall, and their weight loss was slightly higher than those vaccinated with Δ*clpB*. Nonetheless, among the survivors we did not observe any obvious difference of the health status across the three substrains, whether they were vaccinated with LVS or Δ*clpB*.

## Conclusion

To develop and characterize vaccines, the choice of animal models represents an important step that allows the evaluation, screening, and selection of potential vaccine candidates. This approach is even more important for selection of vaccines against diseases such as tularemia, where clinical trials are largely impractical due to the low incidence of the infection in nature. However, the availability of at least three different rat substrains, all named Fischer 344 rats, necessitated the evaluation of *Ft* infection, vaccination, and anti-*Francisella* immune responses by these substrains to understand implications for choices in this infection model. In particular, we focused this study on the evaluation of the substrains readily available from commercial suppliers. The results indicate that, despite minor and detectable differences between the substrains, innate and adaptive immune responses appear to be comparable, susceptibility to aerosol *Ft* infection is similar, and protection by vaccination with both LVS and Δ*clpB* is equivalent among the three rat substrains.

Of note, the Fischer 344 rat model has a general advantage in providing a means to test a wider dynamic range of challenge doses compared to mice or rabbits. However, the present studies were not designed or powered to directly compare relative efficacy of LVS and Δ*clpB*. Nonetheless, accumulated results to date support an emerging picture. Protection against relatively low doses of aerosol *Ft* challenge follow vaccination with Δ*clpB* may be better than that provided by LVS in mice and rats, but protection is roughly equivalent in rabbit studies and when vaccinated rats are challenged with higher doses (Shen et al., 2010; Shoudy et al., 2021; De Pascalis et al., 2022). Any judgement as to which model better reflects outcomes in people obviously awaits clinical studies.

Although these substrains were all derived from the Fischer 344 line, genomic and phenotypic modifications may have occurred while animals were bred independently from each other. These changes may be responsible for some of the subtle differences in patterns we observed, particularly after primary vaccination (Figure 2; Supplementary Figure S1). In these cases, difference in relative gene up-or down-regulation were also seen in non-vaccinated animals, further suggesting some inherent differences. Nonetheless, the differences did not influence the functions of restimulated cells from either naïve or vaccinated animals (Figure 3; Supplementary Figure S2), nor outcomes *in vivo* after aerosol challenge with fully virulent *Ft* SchuS4 (Figure 4; Supplementary Figure S3).

Taken together, therefore, this study demonstrates that the three Fischer 344 substrains are comparable for *Ft* studies. To our knowledge, this is the first comparison of these three Fischer 344 rat substrains for studies of infection and immunity. Perhaps most importantly, the previous finding suggesting stronger protective responses to Δ*clpB* compared to LVS, detected in studies using NHsd rats (De Pascalis et al., 2022), was consistent with the results seen here using DuCrl and IcoCrl rats.

## Data availability statement

The original contributions presented in the study are included in the article/Supplementary material, further inquiries can be directed to the corresponding authors.

## Ethics statement

The animal study was reviewed and approved by Animal Care and Use committee of CBER/FDA (#2015-21), UNMHSC (#19-200938-HSC) MRI Global (AUS 16-33).

## Author contributions

RDP, VB, SE, TW, and HG performed the experiments. RDP, TW, and HG performed statistical analyses. RDP and KE prepared the manuscript, which was reviewed by VB, SE, TW, and HG. All authors contributed to the article and approved the submitted version.

## Funding

This research was partially supported by DoD Chemical and Biological Defense Program through Defense Threat Reduction Agency under contract HDTRA1-16-C-0028.

## Conflict of interest

The authors declare that the research was conducted in the absence of any commercial or financial relationship that could be interpreted as a potential conflict of interest.

## Publisher’s note

All claims expressed in this article are solely those of the authors and do not necessarily represent those of their affiliated organizations, or those of the publisher, the editors and the reviewers. Any product that may be evaluated in this article, or claim that may be made by its manufacturer, is not guaranteed or endorsed by the publisher.

## Supplementary material

Supplementary Figure 1 PBLs from vaccinated rats show patterns of relative gene expression between rat substrains. Fischer rats were vaccinated as indicated. Blood was collected, PBLs were prepared, and semi-quantitative analyses of gene expression were performed as described in Materials and methods and Figure 1. Mean of the Δ*Ct* and standard error of the mean (s.e.m.) for each group were plotted. Brackets indicate significant differences (* = *p* < 0.05). Shown are data from genes of immunological interest analyzed in addition to those shown in Figure 1.

Supplementary Figure 2 Rat leukocytes induce mediator production in co-cultures in similar patterns between substrains. Supernatants collected from in vitro co-cultures, as described in Figure 3, were analyzed for NO (panels **A-D**) and IFN-γ (panels **E-H**). Values shown are the average from 2 – 3 independent experiments of similar design. Error bars indicate standard error of the mean (s.e.m.). *p* values were calculated among rat substrains, within each vaccine group. Bracket indicates significant difference (* = *p* < 0.05).

Supplementary Figure 3 Survival outcomes of naive rats after aerosol challenge with *Ft* demonstrated minimal differences between substrains. Rats were challenged with *Ft* SchuS4 via aerosol, as described in Materials and methods, and monitored for 28 days. Surviving and non-surviving animals were plotted on the Y-axis relative to the number of aerosolized bacteria on the X-axis. The line represents a curve fit to the data and the blue area represents the 95% confidence interval.

Supplementary Figure 4 Body weight of challenged animals reflects infection and survival outcomes. Fischer rats substrains were vaccinated as indicated in Figure 4. Six weeks after vaccination, rats were challenged by aerosol with two different doses of *Ft* SchuS4. Body weight was monitored for the duration of the survival study. Each experiment included five animals per vaccine group per each substrain.

Supplementary Figure 5 Clinical observations of aerosol challenged rats reflect infection and survival outcomes and partially reflect vaccine administration. Fischer rats were vaccinated as indicated in Figure 4. Challenged animals were observed daily for the duration of the survival study, and assigned a clinical score from 0 to 5, with 0 reflecting no outward signs of illness and 5 reflecting an animal found dead.
