# Supplementary figures and images for: In vivo and in vitro immune responses against Francisella tularensis vaccines are comparable among Fischer 344 rat substrains
Source: Front Microbiol. 2023 Jul 13;14:1224480. doi: 10.3389/fmicb.2023.1224480 (PMC10400713; doi:10.3389/fmicb.2023.1224480)

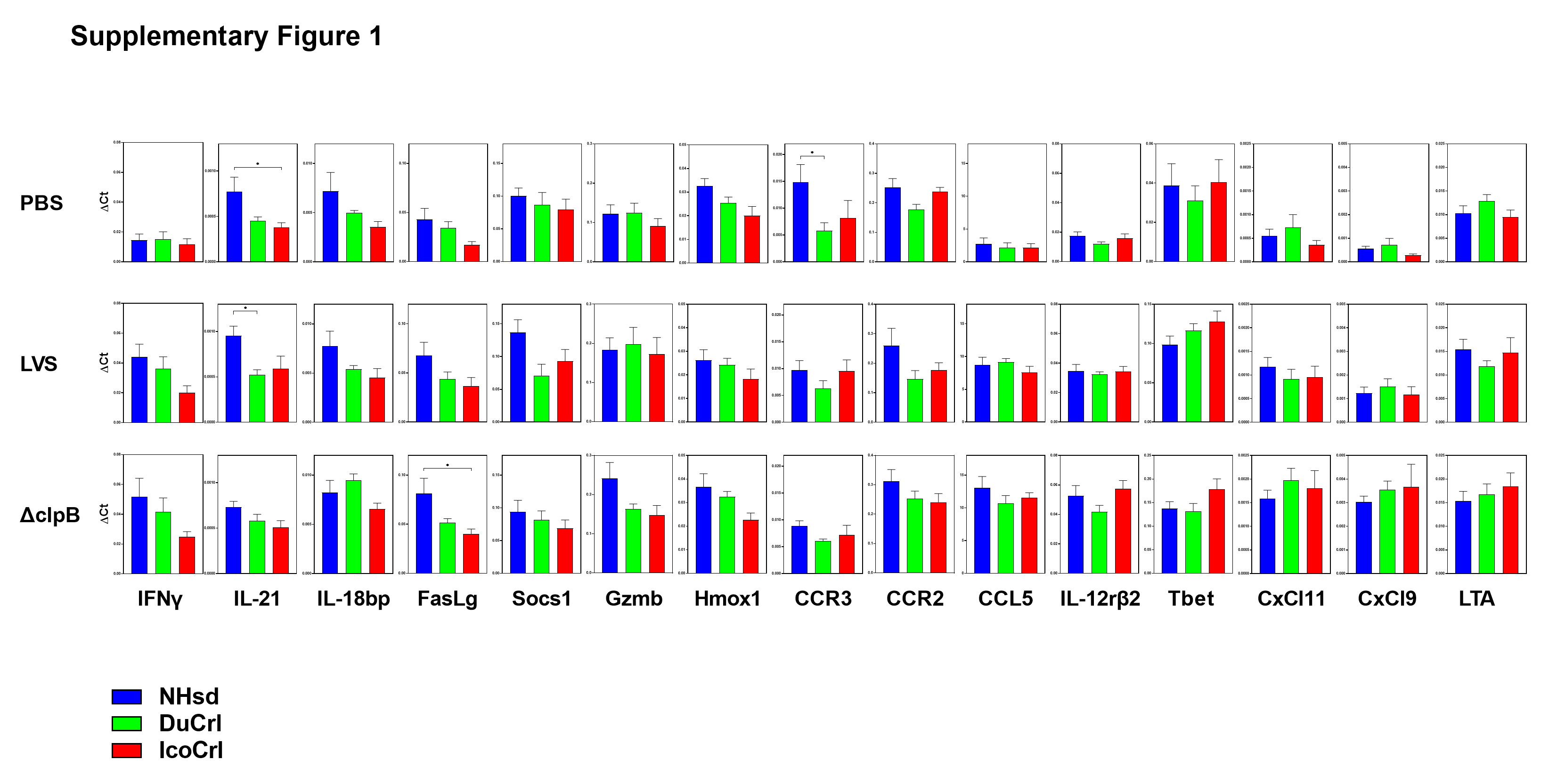

Supplement: Supplementary Figure 1 — PBLs from vaccinated rats show patterns of relative gene expression between rat substrains. Fischer rats were vaccinated as indicated. Blood was collected, PBLs were prepared, and semi-quantitative analyses of gene expression were performed as described in Materials and methods and Figure 1. Mean of the ΔCt and standard error of the mean (s.e.m.) for each group were plotted. Brackets indicate significant differences (* = p < 0.05). Shown are data from genes of immunological interest analyzed in addition to those shown in Figure 1. [file Image_1.TIF]

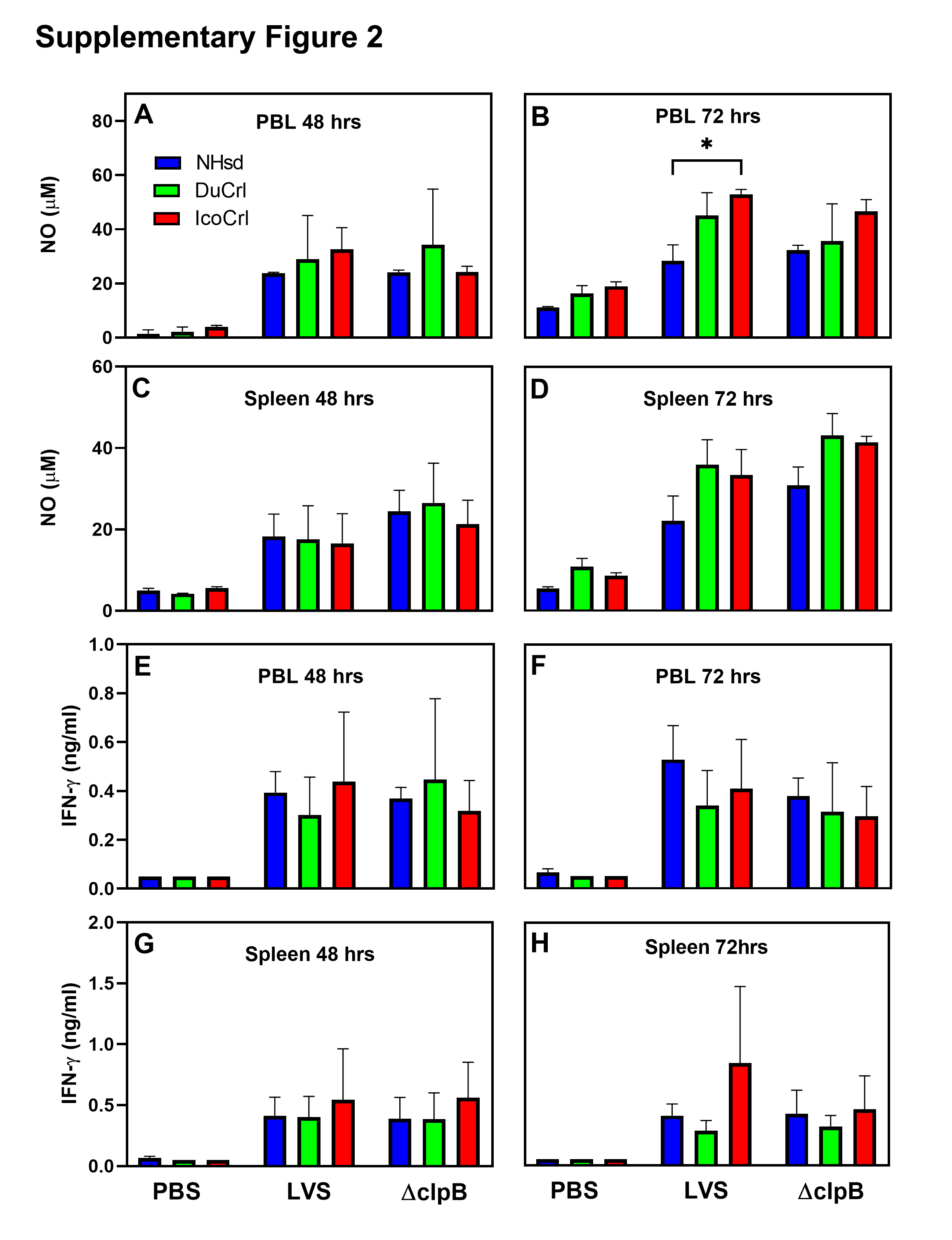

Supplement: Supplementary Figure 2 — Rat leukocytes induce mediator production in co-cultures in similar patterns between substrains. Supernatants collected from in vitro co-cultures, as described in Figure 3, were analyzed for NO (panels A-D) and IFN-γ (panels E-H). Values shown are the average from 2 – 3 independent experiments of similar design. Error bars indicate standard error of the mean (s.e.m.). p values were calculated among rat substrains, within each vaccine group. Bracket indicates significant difference (* = p < 0.05). [file Image_2.TIF]

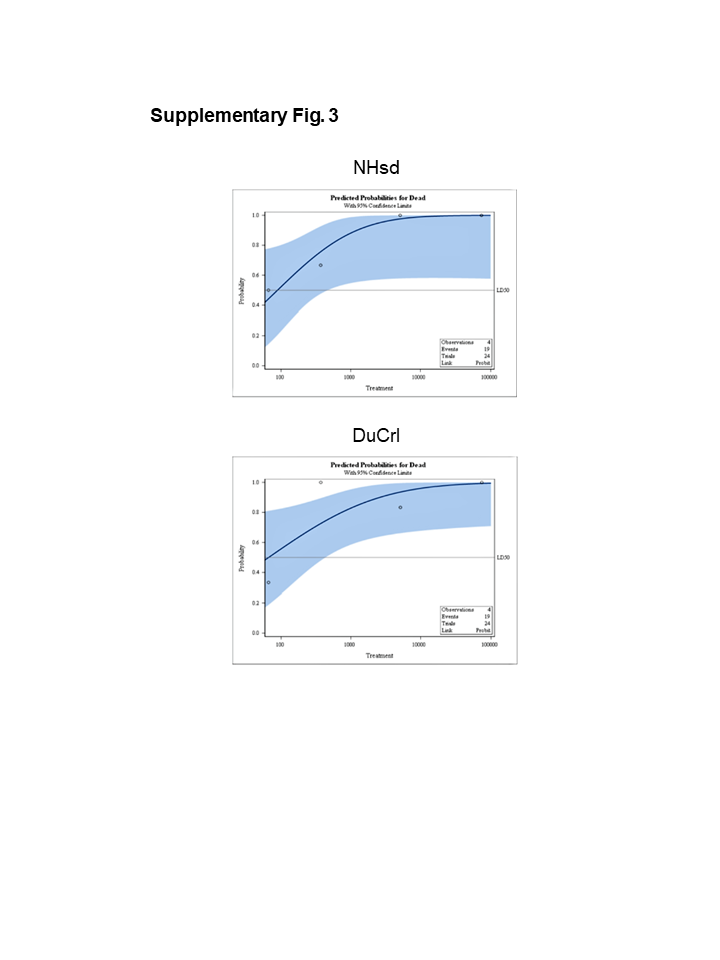

Supplement: Supplementary Figure 3 — Survival outcomes of naive rats after aerosol challenge with Ft demonstrated minimal differences between substrains. Rats were challenged with Ft SchuS4 via aerosol, as described in Materials and methods, and monitored for 28 days. Surviving and non-surviving animals were plotted on the Y-axis relative to the number of aerosolized bacteria on the X-axis. The line represents a curve fit to the data and the blue area represents the 95% confidence interval. [file Image_3.TIF]

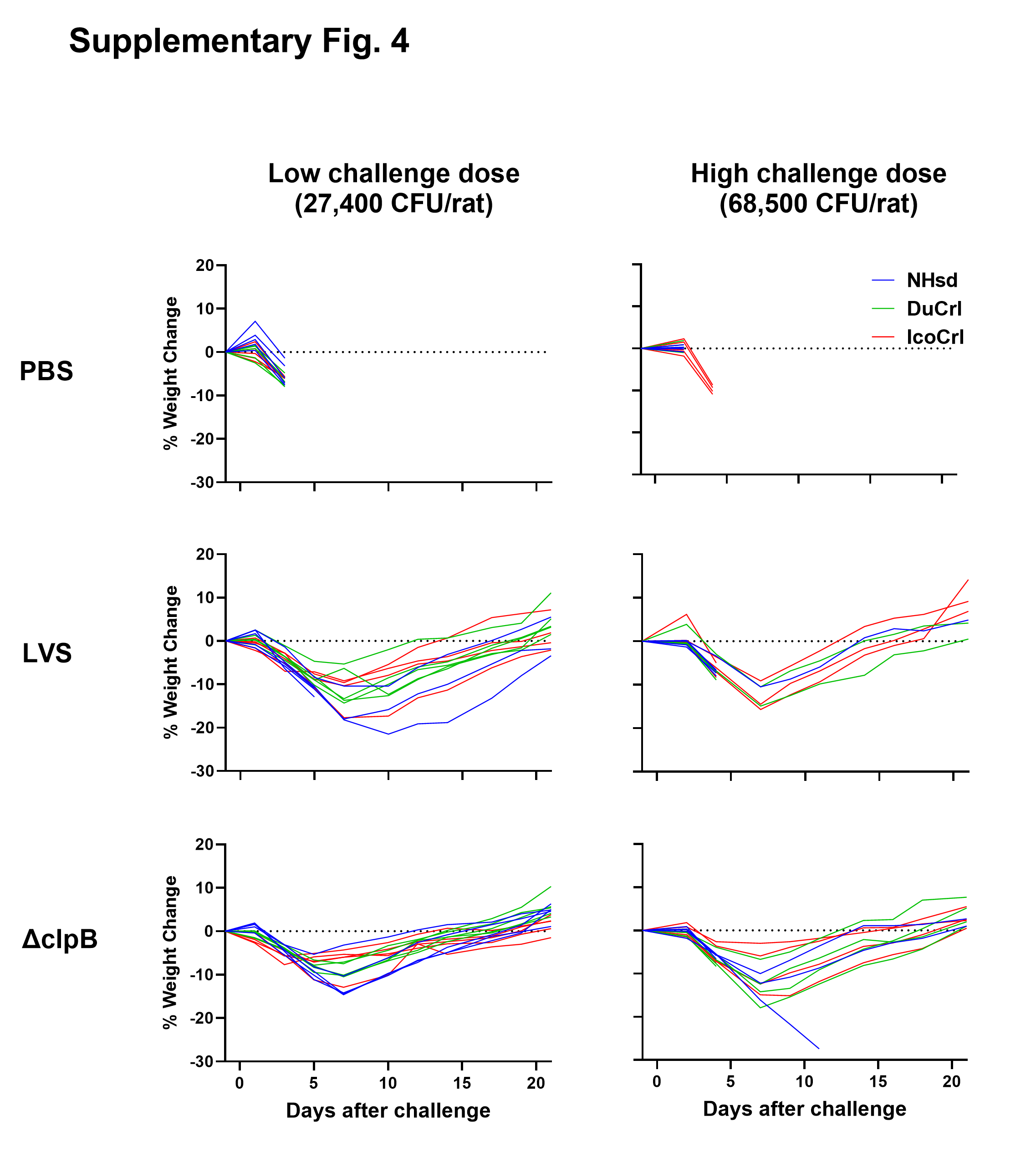

Supplement: Supplementary Figure 4 — Body weight of challenged animals reflects infection and survival outcomes. Fischer rats substrains were vaccinated as indicated in Figure 4. Six weeks after vaccination, rats were challenged by aerosol with two different doses of Ft SchuS4. Body weight was monitored for the duration of the survival study. Each experiment included five animals per vaccine group per each substrain. [file Image_4.TIF]

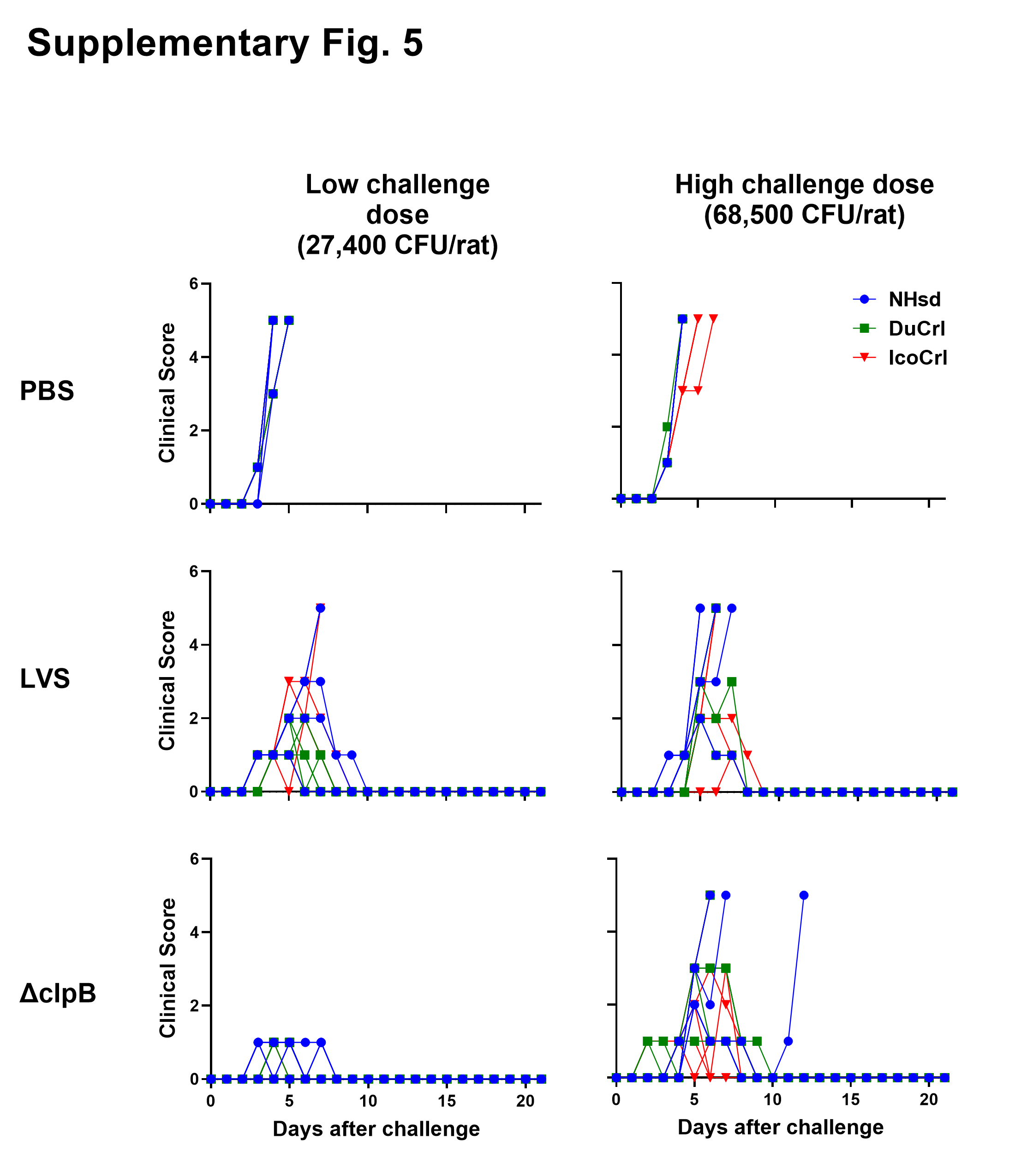

Supplement: Supplementary Figure 5 — Clinical observations of aerosol challenged rats reflect infection and survival outcomes and partially reflect vaccine administration. Fischer rats were vaccinated as indicated in Figure 4. Challenged animals were observed daily for the duration of the survival study, and assigned a clinical score from 0 to 5, with 0 reflecting no outward signs of illness and 5 reflecting an animal found dead. [file Image_5.TIF]
