# Supplementary material for: In vivo and in vitro immune responses against Francisella tularensis vaccines are comparable among Fischer 344 rat substrains
Source: Front Microbiol. 2023 Jul 13;14:1224480. doi: 10.3389/fmicb.2023.1224480 (PMC10400713; doi:10.3389/fmicb.2023.1224480)
Supplement: Supplementary file 6 [file Data_Sheet_1.PDF]

Supplementary Table 1. Relative gene expression of immune-related factors in PBLs two weeks after vaccination.

	NHsd		DuCrl		IcoCrl	
	LVS	ΔclpB	LVS	ΔclpB	LVS	ΔclpB
IFN-γ	3.0 ± 0.6	3.5 ± 0.9	2.4 ± 0.5	2.8 ± 0.6	1.7 ± 0.4	2.1 ± 0.3
NOS2	1.3 ± 0.2	1.4 ± 0.3	1.1 ± 0.3	1.6 ± 0.4	1.2 ± 0.4	1.4 ± 0.3
IL-21	1.2 ± 0.1	1.0 ± 0.08	1.2 ± 0.1	1.3 ± 0.2	1.6 ± 0.4	1.3 ± 0.2
IL-18bp	1.1 ± 0.2	1.1 ± 0.2	1.1* ± 0.07	1.9* ± 0.1	1.3* ± 0.3	1.8* ± 0.2
FASLG	1.6 ± 0.3	1.9 ± 0.4	1.3 ± 0.2	1.5 ± 0.1	2.2 ± 0.5	2.4 ± 0.3
SOCS1	1.4 ± 0.2	0.9 ± 0.2	0.8 ± 0.2	0.9 ± 0.2	1.2 ± 0.2	0.9 ± 0.2
GZMB	1.5 ± 0.3	2.0 ± 0.3	1.6 ± 0.3	1.3 ± 0.1	1.9 ± 0.5	1.6 ± 0.3
HMOX1	0.8 ± 0.1	1.1 ± 0.2	1.0* ± 0.1	1.3* ± 0.1	0.9 ± 0.2	1.1 ± 0.1
CCR3	0.7 ± 0.1	0.6 ± 0.06	1.1 ±0.2	1.0 ± 0.07	1.2 ±0.3	0.9 ± 0.2
CCR2	1.0 ± 0.2	1.2 ± 0.2	0.8* ± 0.2	1.4* ± 0.1	0.7 ± 0.1	1.0 ± 0.1
CCR5	2.0 ± 0.2	2.1 ± 0.2	1.9* ± 0.2	2.6* ± 0.2	2.0 ± 0.2	2.2 ± 0.1
CCL5	3.2* ± 0.4	4.8* ± 0.6	4.3 ± 0.3	4.9 ± 0.6	3.5* ± 0.4	5.4* ± 0.4
IL-12rβ2	2.0* ± 0.3	3.0* ± 0.4	2.7* ± 0.1	3.5* ± 0.4	2.2* ± 0.2	3.6* ± 0.4
TBET	2.5* ± 0.3	3.5* ± 0.4	3.7 ± 0.3	4.2 ± 0.6	3.2* ± 0.3	4.4* ± 0.5
CXCR6	2.1 ± 0.4	2.4 ± 0.3	1.9 ± 0.2	2.5 ± 0.3	1.8 ± 0.2	1.9 ± 0.2
CXCl11	2.2 ± 0.4	2.9 ± 0.3	1.3* ± 0.3	2.7* ± 0.4	2.7* ± 0.7	5.0* ± 1.1
CXCl9	2.2* ± 0.5	5.4* ± 0.4	2.1* ± 0.5	4.9* ± 0.5	4.2* ± 1.2	13.2* ± 3.4
LTA	1.5 ± 0.2	1.5 ± 0.2	0.9* ± 0.1	1.3* ± 0.2	1.5 ± 0.3	1.9 ± 0.3
IL-2ra	0.6* ± 0.1	0.5* ± 0.04	0.7 ± 0.1	0.6 ± 0.1	1.1 ± 0.3	0.7 ± 0.1

Gene expression analyses were performed using PBLs as shown in Fig. 2 and Table 1. Fold changes were calculated in comparison to mean of naïve cells from the matching rat substrain. Shown are the means of fold change ± standard error of the mean (s.e.m.) calculated from 7 - 10 animal per group. Within each rat substrain group, * indicates significant differences between LVS and *ΔclpB* vaccines ($P < 0.05$).
