# Supplementary material for: In vivo and in vitro immune responses against Francisella tularensis vaccines are comparable among Fischer 344 rat substrains
Source: Front Microbiol. 2023 Jul 13;14:1224480. doi: 10.3389/fmicb.2023.1224480 (PMC10400713; doi:10.3389/fmicb.2023.1224480)
Supplement: Supplementary file 7 [file Data_Sheet_2.pdf]

Supplementary Table 2. Relative gene expressionof immune-related factors in rat PBLs and splenocytes recovered from co-cultures

PBL

	NHsd		DUCrl		IcoCrl	
	LVS	*ΔclpB*	LVS	*ΔclpB*	LVS	*ΔclpB*
CCL5	3.1* ± 0.5	5.5* ± 0.2	3.4* ± 1.2	7.6* ± 0.9	2.6* ± 0.6	6.3* ± 0.6
CCR3	1.05 ± 0.02	1.8 ± 0.2	1.9 ± 1.1	4.4 ± 3.3	0.9 ± 0.1	1.9 ± 0.9
CCR5	0.9 ± 0.1	1.0 ± 0.2	0.8 ± 0.3	0.9 ± 0.03	0.6 ± 0.1	0.7 ± 0.3
CXCL9	14.1 ± 11.2	5.1 ± 1.5	10.1 ± 8.7	16.7 ± 15.7	10.9 ± 6.0	9.3 ± 0.3
CXCR6	2.2 ± 0.4	3.6 ± 0.9	2.8 ± 0.8	4.4 ± 1.0	2.3* ± 0.4	6.9* ± 1.1
FASLG	1.8* ± 0.03	2.0* ± 0.02	2.3 ± 0.1	2.9 ± 0.3	1.3 ± 0.6	1.5 ± 0.6
GZMB	2.4 ± 0.6	2.3 ± 0.2	1.7 ± 0.1	1.8 ± 0.4	4.2 ± 1.1	3.0 ± 1.6
HMOX1	8.9 ± 6.1	2.7 ± 0.02	5.3 ± 4.9	7.2 ± 6.7	2.6 ± 0.3	1.9 ± 1.1
IFN-γ	4.9 ± 0.8	4.6 ± 1.5	6.0 ± 2.4	7.9 ± 4.5	11.3 ± 0.8	15.5 ± 9.3
IL-2RA	3.4* ± 0.03	2.2* ± 0.1	4.2 ± 1.7	3.3 ± 1.4	3.3 ± 1.8	2.5 ± 1.2
IL-12rβ2	3.2 ± 0.3	3.1 ± 0.2	3.5 ± 1.3	4.0 ± 1.2	3.5 ± 0.2	2.9 ± 0.7
IL-18bp	4.4 ± 1.5	2.7 ± 0.7	3.4 ± 2.4	4.4 ± 3.3	3.4 ± 2.2	3.6 ± 1.5
IL-21	30.1 ± 8.9	18.7 ± 2.5	23.5 ± 2.9	16.4 ± 5.7	53.1 ± 33.2	31.8 ± 1.6
LTA	10.0 ± 2.7	6.6 ± 0.9	8.4 ± 2.8	5.9 ± 1.5	10.7 ±2.8	10.1 ± 1.0
NOS2	10.4 ± 2.4	10.3 ± 6.9	3.6 ± 1.9	6.1 ± 4.4	16.9 ± 2.8	16.6 ± 12.7
SOCS1	1.9 ± 0.9	1.7 ± 0.4	1.4 ± 0.5	1.2 ± 0.4	3.5 ± 1.6	4.5 ± 2.6
TBET	1.9 ± 0.8	2.4 ± 0.4	1.9 ± 0.7	1.5 ± 1.7	2.4 ± 0.9	3.8 ± 2.2
IL-6	0.5 ± 0.2	0.4 ± 0.08	0.5 ± 0.3	0.3 ± 0.2	0.9 ± 0.5	1.0 ± 0.5
CSF2	1.6 ± 1.0	0.9 ± 0.3	1.3 ± 0.9	0.9 ± 0.8	2.8 ± 1.0	1.8 ± 0.7
CXCL11	0.5 ± 0.01	0.3 ± 0.1	0.2 ± 0.03	0.2 ± 0.1	1.4 ± 1.3	1.5 ± 1.4
CCR2	1.3 ± 0.09	2.2 ± 0.3	2.0 ± 0.2	2.8 ± 0.5	2.0 ± 0.4	3.6 ± 0.4
TNFα	1.6 ± 0.4	1.5 ± 0.3	1.0 ± 0.2	0.8 ± 0.2	2.3 ± 0.5	2.4 ± 0.6
PRF1	1.7 ± 0.4	2.1 ± 0.02	1.6 ± 0.3	1.5 ± 0.5	2.1 ± 0.02	2.3 ± 1.5

Spleen

	NHsd		DuCrl		IcoCrl	
	LVS	*ΔclpB*	LVS	*ΔclpB*	LVS	*ΔclpB*
CCL5	1.3* ± 0.2	3.0* ± 0.2	1.7 ± 0.01	4.3 ± 1.3	1.3* ± 0.01	3.4* ± 0.3
CCR3	0.8 ± 0.1	1.4 ± 0.3	0.9 ± 0.06	1.5 ± 0.3	0.5 ± 0.05	1.6 ± 0.6
CCR5	1.6* ± 0.04	2.4* ± 0.1	1.8 ± 0.4	2.4 ± 1.0	1.6 ± 0.3	1.8 ± 0.5
CXCL9	4.0 ±0.9	6.6 ± 1.8	5.6 ± 2.2	7.5 ± 4.5	4.1 ± 1.8	4.2 ± 0.3
CXCR6	0.9 ± 0.2	1.9 ± 0.4	1.1 ± 0.04	1.6 ± 0.4	1.0 ± 0.03	1.3 ± 0.03
FASLG	1.7* ± 0.08	2.6* ± 0.3	1.9 ± 0.1	2.8 ± 0.3	2.1 ± 0.1	2.3 ± 0.05

GZMB	3.6 ± 1.3	4.5 ± 1.3	2.9* ± 0.02	5.3* ± 0.4	8.0 ± 0.01	8.3 ± 0.1
HMOX1	1.3 ± 0.4	2.0 ± 0.1	2.4 ± 0.9	4.1 ± 1.9	1.7 ± 0.7	2.3 ± 1.0
IFN-γ	13.1 ± 3.9	14.8 ± 3.1	18.0 ± 6.5	19.6 ± 12.9	21.6 ± 5.3	16.9 ± 8.4
IL-2RA	1.8 ± 0.08	2.0 ± 0.3	3.0 ± 0.8	2.2 ± 0.9	3.5 ± 0.6	2.0 ± 0.3
IL-12rβ2	2.5* ± 0.3	3.5* ± 0.3	3.7 ± 0.6	3.9 ± 1.6	5.0 ± 0.5	4.1 ± 0.8
IL-18bp	1.5 ± 0.07	2.2 ± 0.3	1.6 ± 0.07	2.1 ± 0.8	1.7 ± 0.06	1.8 ± 0.01
IL-21	12.8 ± 2.4	11.9 ± 1.2	30.7 ± 3.4	19.8 ± 6.6	42.7 ± 2.8	14.8 ± 2.3
LTA	6.5 ± 1.6	7.8 ± 2.4	10.9 ± 3.7	8.9 ± 5.2	13.9 ± 3.0	9.9 ± 2.1
NOS2	2.6 ± 0.5	4.2 ± 1.5	3.6 ± 1.6	4.8 ± 2.6	4.3 ± 1.3	4.2 ± 0.2
SOCS1	2.8 ± 0.4	3.6 ± 0.4	3.8 ± 0.04	2.4 ± 0.6	3.9 ± 0.03	2.9 ± 0.8
TBET	1.7 ± 0.03	2.2 ± 0.1	2.1 ± 0.04	2.1 ± 0.5	2.2 ± 0.03	2.1 ± 0.1
IL-6	0.7 ± 0.3	0.6 ± 0.2	0.5 ± 0.09	0.4 ± 0.06	0.5 ± 0.07	0.5 ± 0.2
CSF2	1.2 ± 0.4	0.8 ± 0.2	1.2* ± 0.01	0.6* ± 0.07	1.3 ± 0.01	0.6 ± 0.2
CXCL11	1.6 ± 0.5	3.5 ± 1.6	1.9 ± 0.4	1.9 ± 0.6	1.6 ± 0.3	1.6 ± 0.6
CCR2	1.7 ± 0.5	2.9 ± 0.8	2.0 ± 0.5	2.9 ± 1.3	1.8 ± 0.4	2.8 ± 0.2
TNFα	1.5 ± 0.4	1.8 ± 0.6	1.5 ± 0.2	1.2 ± 0.3	1.8 ± 0.2	1.6 ± 0.02
PRF1	1.4 ± 0.2	1.6 ± 0.2	1.0 ± 0.04	1.5 ± 10.1	2.1 ± 0.04	2.4 ± 0.4

Gene expression analyses using cells recovered from *in vitro* co-cultures after 48 hours were performed as described in Material and Methods and Table 2. Fold changes were calculated in comparison to naïve cells and means of fold change ± standard error of the mean (s.e.m.) were calculated from 2 or 3 independent experiments. Within each rat substrain group, * indicate significant differences between LVS and *ΔclpB* vaccines ($P < 0.05$).
