# Supplementary material for: In vivo and in vitro immune responses against Francisella tularensis vaccines are comparable among Fischer 344 rat substrains
Source: Front Microbiol. 2023 Jul 13;14:1224480. doi: 10.3389/fmicb.2023.1224480 (PMC10400713; doi:10.3389/fmicb.2023.1224480)
Supplement: Supplementary file 8 [file Data_Sheet_3.docx]

**Supplementary Figure 1**. **PBLs from vaccinated rats** **show patterns of relative gene expression between rat substrains**. Fischer rats were vaccinated as indicated. Blood was collected, PBLs were prepared, and semi-quantitative analyses of gene expression were performed as described in Materials and Methods and Figure 1. Mean of the ΔCt and standard error of the mean (s.e.m.) for each group were plotted. Brackets indicate significant differences (* = *P* < 0.05). Shown are data from genes of immunological interest analyzed in addition to those shown in Figure 1.

**Supplementary Figure 2. Rat leukocytes induce mediator production in co-cultures in similar patterns between substrains**. Supernatants collected from *in vitro* co-cultures, as described in Figure 3, were analyzed for NO (panels A-D) and IFN-γ (panels E-H). Values shown are the average from 2 – 3 independent experiments of similar design. Error bars indicate standard error of the mean (s.e.m.). *P* values were calculated among rat substrains, within each vaccine group. Bracket indicates significant difference (* = *P* < 0.05).

**Supplementary Figure 3. Survival outcomes of naive rats after aerosol challenge with *Ft* demonstrated minimal differences between substrains.** Rats were challenged with *Ft* SchuS4 via aerosol, as described in Materials and Methods, and monitored for 28 days. Surviving and non-surviving animals were plotted on the Y-axis relative to the number of aerosolized bacteria on the X-axis. The line represents a curve fit to the data and the blue area represents the 95% confidence interval.

**Supplementary Figure 4**. **Body weight of challenged animals reflects infection and survival outcomes.** Fischer rats substrains were vaccinated as indicated in Figure 4. Six weeks after vaccination, rats were challenged by aerosol with two different doses of *Ft* SchuS4. Body weight was monitored for the duration of the survival study. Each experiment included five animals per vaccine group per each substrain.

**Supplementary Figure 5.** **Clinical observations of aerosol challenged rats reflect infection and survival outcomes and partially reflect vaccine administration**. Fischer rats were vaccinated as indicated in Figure 4. Challenged animals were observed daily for the duration of the survival study, and assigned a clinical score from 0 to 5, with 0 reflecting no outward signs of illness and 5 reflecting an animal found dead.
